# A New Perspective on the Co-Transmission of Plant Pathogens by Hemipterans

**DOI:** 10.3390/microorganisms11010156

**Published:** 2023-01-07

**Authors:** Cecilia Tamborindeguy, Fernando Teruhiko Hata, Rúbia de Oliveira Molina, William Mário de Carvalho Nunes

**Affiliations:** 1 Department of Entomology, Texas A&M University, College Station, TX 77843, USA; 2 Department of Agronomy, Universidade Estadual de Maringá (UEM), Colombo Avenue, 5790—Zona 7, Maringá 87020-900, Brazil; 3 Paraná Rural Development Institute—IAPAR—EMATER (IDR-Paraná), Celso Garcia Cid, Km 375, Londrina 86047-902, Brazil

**Keywords:** virus, bacteria, aphid, whitefly, psyllid, circulation, receptor, competition, synergism

## Abstract

Co-infection of plants by pathogens is common in nature, and the interaction of the pathogens can affect the infection outcome. There are diverse ways in which viruses and bacteria are transmitted from infected to healthy plants, but insects are common vectors. The present review aims to highlight key findings of studies evaluating the co-transmission of plant pathogens by insects and identify challenges encountered in these studies. In this review, we evaluated whether similar pathogens might compete during co-transmission; whether the changes in the pathogen titer in the host, in particular associated with the co-infection, could influence its transmission; and finally, we discussed the pros and cons of the different approaches used to study co-transmission. At the end of the review, we highlighted areas of study that need to be addressed. This review shows that despite the recent development of techniques and methods to study the interactions between pathogens and their insect vectors, there are still gaps in the knowledge of pathogen transmission. Additional laboratory and field studies using different pathosystems will help elucidate the role of host co-infection and pathogen co-transmission in the ecology and evolution of infectious diseases.

## 1. Introduction

Hemipteran insects are the primary vectors of plant pathogens; they are responsible for transmitting 72% of plant viruses with a known vector [1]. Because of the central role of transmission in the spread of many viruses and bacteria causing devastating plant diseases, pathogen transmission by hemipterans has been widely studied. Historically, a large body of literature has been dedicated to the study of virus transmission by aphids because of the economic importance of the pathogens they transmit, the large number of aphid vector species, as well as critical features of aphid biology that make them amenable organisms to study. In recent decades, advances in tools and techniques available for scientists as well as the increasing spread of devastating diseases caused by different plant pathogens have led to the study of a multitude of pathogen–vector systems including non-hemipteran vectors such as thrips, mites, or beetles [2,3,4,5].

The transmission process includes acquisition of the pathogen from an infected source by the arthropod vector and its inoculation into a new host. Virus transmission is classified into four mechanisms depending on where the virus attaches or localizes in the vector, the persistence of the virus in the vector, and whether or not the virus replicates in the vector [6]. Non-persistent and semi-persistent transmissions are characterized by short retention of the virus within the vector, virus attachment to the mouthparts or the foregut of the vector, and absence of virus replication within the vector [7]. In the persistent circulative and persistent propagative mechanisms, the vector remains viruliferous for a more extended period as the virus enters and circulates throughout the arthropod body [1,8]. The persistent propagative transmission is the only mechanism in which the virus replicates in the vector. These mechanisms are also used to describe bacterial transmission; however, bacteria do not necessarily need to invade the vector cells to replicate.

While studies generally focus on the transmission of a single pathogen, co-infections of plants by different pathogens are common in nature and occur in diverse plant–vector–pathogen systems [9,10]. Therefore, co-transmission could be common in nature and not an exception. From the complex interaction between plants and pathogens, a synergistic or antagonist interaction may occur as a result of the spatiotemporal order of infection [11,12,13]. Synergism can manifest itself by an increase in viral replication, or the cooperation and coexistence between members of the viral complex, affecting both or at least one of the viruses involved. The resulting symptoms that develop in the host are greater than the sum of the individual effects [11,14]. For example, due to the synergism between both viruses, the co-infection of cowpea severe mosaic virus (CSMV) and cucumber mosaic virus (CMV) results in more severe symptoms, including dwarfism along with extreme mosaic, leaf deformation, and in some cultivars general necrosis [15]. Similarly, cassava crops suffer more severe damage when two begomoviruses—the African cassava mosaic virus (ACMV) and the Ugandan strain of East African cassava mosaic virus (EACMV-UG)—co-infect [16]. Therefore, co-infections can affect the ecology of the diseases caused by a pathogen as the presence of other pathogens can influence the pathogen load, the expression of virulence genes, the distribution of the pathogen within plants, and its transmission [17]. Similarly, the co-transmission of pathogens also occurs as a result of the vector acquiring two or more pathogens simultaneously or sequentially from different feedings. Indeed, pathogen transmission often relies on specific tissue tropism within the vector, interaction with vector proteins, and manipulation of the insect’s immunity. Therefore, co-transmission could result in decreased transmission, for example, if there is competition for specific vector proteins involved in transmission such as stylin or cyclophilin [18,19,20], or induction or repression of the vector immune defenses [21,22]. Alternatively, it could result in increased transmission if one pathogen facilitates the transmission of the other by acting as a helper virus [23]. Excellent studies have previously reported or reviewed several examples of co-transmission of plant pathogens, such as [24] or [25]. Therefore, the present review is not a comprehensive review of all the co-transmission studies published, just of a few which allow us to showcase new findings and identify challenges encountered in the study of co-transmission as well as future perspectives. Because of the importance of phloem-feeding hemipterans as vectors, a vast majority of the articles reviewed here focus on these insects. Moreover, plant co-infections can be spread by vectors that acquired the pathogens from mixed infected plants, successively from separate plants, or by various vectors that each acquired a different pathogen. Many of the published studies evaluated the epidemiological implication of the co-infection and co-transmission of pathogens using groups of vectors instead of single individuals because in nature several insects might infect the plants. When possible, in this review, we focus on the studies using single insects for transmission.

## 2. Are Closely Related Pathogens More Likely to Compete during Co-Transmission?

It could be tempting to assume that closely related pathogens have an antagonistic interaction as they compete for similar transmission sites and vector proteins involved in transmission such as receptors. Certainly, closely related viruses that share a similar transmission mechanism and vector species also share similar structural and non-structural proteins. Then, competition during transmission is likely to occur, at least for non-persistently transmitted viruses. This transmission mechanism involves few vector and virus proteins [7]. However, the study of the co-infection and co-transmission of different strains of non-persistently transmitted viruses reveals that their interactions with plants and vectors are not so simple (discussed below) and that these viruses do not always compete during co-transmission. It is therefore evident that simple competition might also not be the case for persistently transmitted viruses which interact with a diversity of vector proteins. For example, luteovirus transmission by cereal aphids was determined to be a polygenic trait, with genes acting in an additive manner [26,27,28].

### 2.1. Multipartite Viruses: Similar Virus Particles with a Similar Transmission Mechanism

Multipartite viruses have a segmented genome and each viral segment is encapsidated in an individual capsid [29]. Therefore, host co-infection is a requirement for these viruses. Some examples of virus families which have multipartite species infecting plants of agricultural interest are *Closteroviridae*, *Geminiviridae*, *Nanoviridae*, *Potyviridae,* and *Rhabdoviridae* [30]. Because of the need for co-infection with multiple genome segments for multipartite viruses, a high viral load or multiplicity of infection (MOI) could be needed to achieve infection even though there is no experimental data indicating that transmission with high MOI between hosts [31]. Further, the majority of these viruses are transmitted by insects, and their transmission, in fact, happens at low MOI [31]. Then, hypothetically, a higher vector population is needed for transmitting the complete genetic information to initiate an infection.

While it could be argued that the transmission of multipartite viruses does not qualify as viral co-transmission of different viruses, the study of their transmission can shed light on several challenges and questions faced during the study of virus co-transmission. Successful host infection relies on the presence of several of the genome segments, and while in nature plant inoculation could be achieved by different insect individuals transmitting some of the segments, a single insect can transmit the virus and cause infection [31].

The ability of single insect vectors to transmit multipartite viruses is not surprising because all segments are encapsidated by the same capsid proteins, thus an insect that can transmit one segment should transmit all of them. Indeed, different segments were observed co-localizing in the same aggregates inside the vector gut cells [32] supporting the existence of a common transmission route for the different segments. A fascinating discovery made for nanoviruses and other multipartite viruses is the existence of the “setpoint genome formula” corresponding to the frequency of each segment in the host [31,33]. Surprisingly, this formula changes depending on the host plant [33,34], and this modification of the relative frequency of each segment is believed to allow the virus to adapt to different environments similar to gene expression regulation in cells. Further, it was determined that the virus formula of faba bean necrotic stunt virus (FBNSV; *Nanoviridae*) in its insect vector is different from that in the plant host [35]. This finding is unexpected because these viruses are transmitted in a circulative non-propagative manner by aphids and as previously established, all particles are in theory identical. In the case of FBNSV, this selection of the virus segments occurs in the gut of the aphid vector [35]. Different hypotheses have been advanced to explain this change in genome formula, including the possibility that these viruses replicate in the vector for a short period of time, differences in stability of the different particles, or differences in affinity to specific receptors [35]. The last two hypotheses would be the consequence of small differences in each particle structure as they encapsidate different genome fragments. Indeed, differences in the capsid-RNA contacts among brome mosaic virus (BMV; *Bromoviridae*) particles affecting their stability were identified [36].

### 2.2. Co-Transmission of Different Strains or Isolates of a Virus

Transmission by vectors is considered to be a virus population bottleneck [37,38]. For example, citrus plants can become infected with multiple strains of citrus tristeza virus (CTV; *Closteroviridae*) through the transmission by several aphids, with grafting, mutation, and recombination occurring during the life of the plant. These different CTV isolates can be separated by aphid transmission because an individual aphid only transmits a few isolates each time [39,40,41,42]. Indeed, the genetic bottleneck was determined for CTV transmitted by *Toxoptera citricida* Kirkaldy (Hemiptera: Aphididae) during aphid acquisition [43]. Similarly, a genetic bottleneck during mite acquisition was also determined for wheat streak mosaic virus (WSMV; *Potyviridae*) strains transmitted by *Aceria tosichella* Keifer (Acari: Eriophyidae) [44]. Both viruses are transmitted in a semi-persistent manner and for both viruses, while the vectors were able to transmit several strains at the same time, few instances of co-transmission were observed. In both cases, it was hypothesized that the preferential acquisition of a specific strain resulted from the spatial separation of the virus strains within the co-infected plant. Indeed, when a host is pre-infected with a virus, it is often protected against a secondary infection (superinfection) by a different isolate of the same or a closely related virus. This phenomenon, called cross-protection, is used to protect crops from severe isolates by pre-infecting them with mild isolates of a virus; this is commonly used to protect citrus from CTV. Cross-protection results in decreased replication and thus titer of the second challenging virus, exclusion from cells already infected by one of the viruses, and spatial separation within the plant.

A genetic bottleneck was reported for CMV also. Individual *Myzus persicae* Sulzer (Hemiptera: Aphididae) and *Aphis gossypii* Glover (Hemiptera: Aphididae) transmitted several CMV mutants from leaves inoculated with twelve CMV mutants, but only some of the mutants were transmitted at the same time by individual aphids [45]. On average, three mutants were transmitted by each individual aphid and some mutants showed differential transmission efficiency depending on the vector species. The mutations studied did not alter the sequence of the virus coat protein; this is the unique viral determinant of CMV transmission by aphids [46]. However, slight changes in the viral particle structure could exist depending on the RNA–protein interactions leading to differences in the stability or retention of the particles as previously discussed for multipartite viruses. CMV is transmitted in a non-persistent manner, and in the case of the mutants, the transmission bottleneck event was linked to the inoculation of the viral mutants, not their acquisition.

The co-transmission of different potato virus Y (PVY; *Potyviridae*) strains by aphids was also investigated by several laboratories [47,48,49,50]. Despite using different virus isolates, vector clones, and host plants, these studies concluded that a single aphid could transmit more than one PVY strain, either acquired from a single plant or following sequential acquisition from different infected material. In both cases, the transmission efficiency was reduced when compared to the single transmission, which is expected if there is competition among viruses. The lower transmission efficiency when the strains were acquired from co-infected plants could be linked to the spatial separation of the viruses within the host [51,52,53], or differences in strain titer which depend on different factors including the strain, the time elapsed since infection, the plant species, or plant variety [54]. However, PVY strains were shown to be separated spatially in plants sometimes but not always, and no correlation between PVY titer and transmission was identified [55].

A lower PVY transmission rate after sequential acquisition could be linked to the short persistence of the viruses within the vector or competition for retention sites. While interference was not observed by Syller and Grupa [56], the study performed by KatisCarpenter and Gibson [57] determined that the acquisition of PVY^N^ negatively affected the transmission of PVY^O^, irrespective of the acquisition order. Similarly, PVY^NTN^ and PVY^N:O^ outcompeted PVY^O^ [48,50]. These findings would suggest differences in retention (one strain may be released more efficiently than the other from the stylet) or affinity for retention sites in the aphid stylet (one strain could bind the stylet more efficiently than the other or different strains could bind to different sites within the stylet). Potyviruses are non-persistently transmitted viruses and rely on a helper component for their transmission, so changes in transmission efficiency could result from the interaction between the helper component and the aphid stylet or between the helper component and the virus particles. Recent work studying the interactions between the aphid stylet, the PVY virus particles, and the helper component determined that PVY^NTN^ and PVY^N:O^ are transmitted more efficiently by the PVY^O^ helper component than by their own and that PVY^NTN^ binds preferentially to the stylet than PVY^O^ [58]. These findings could help explain, at least partially, the co-transmission results.

### 2.3. Co-Transmission of Different Virus Species Sharing a Similar Transmission Mechanism

Different virus species that share vectors and which are transmitted following the same mechanism might also compete for similar receptor sites or receptor molecules in the vector [37]. Depending on the degree of similarity between the two virus species and the diversity of receptors in the vector, interactions between different viruses during the transmission might occur. In general, viruses with high genetic similarity are expected to have an antagonistic relationship while low genetic similarity among the viruses could result in synergistic effects [59]. Several studies evaluated the co-transmission of very similar as well as more diverse viruses.

Several studies reported competition during transmission between viruses assigned to the same genus. For example, co-transmission of potyviruses or criniviruses can result in decreased transmission [57,60,61]. In two of these studies, the competition between the viruses in the host plants was reduced by using recipient plants that can only be infected with one of the viruses or by performing the acquisition of purified virus particles from membrane sachets. Therefore, competition between the viruses probably occurred within the vector, and the results suggest that viruses might share common sites on the vector and interfere with each other during their transmission.

The co-transmission of circulative viruses has also been studied but to our knowledge to a lesser degree. Circulative viruses move through the insect gut, transit in the hemolymph, and cross the salivary glands, but they do not replicate in the vector. These transports rely on interactions with several vector proteins [8]. Based on the suite of proteins encoded by an insect, differences in vector competence exist among individuals of the same species. The diversity of the vector proteins involved in this process has the potential to exacerbate or reduce the competition between pathogens depending on the specificity of the interactions and the existence of overlapping proteins with similar roles.

MAV and PAV are two species of the barley yellow dwarf virus (BYDV; *Tombusviridae*) complex. They are serologically related but not identical, they cross-protect against each other in plants, and they compete for transmission by *Sitobion avenae* Fabricius (Hemiptera: Aphididae) [62]. In sequential acquisition experiments as well as following virus microinjection into the hemolymph, PAV transmission was decreased [63]. Because plants can be co-infected if they are co-inoculated and the cross-protection only occurs if one species is inoculated at least four days prior to the second [64], the authors concluded that the competition between the viruses occurred in the aphid and not during infection after the transmission and that the viruses probably competed for receptors to cross the salivary gland barrier [63]. However, *Rhopalosiphum padi* L. (Hemiptera: Aphididae) was able to transmit PAV but not MAV when it was fed on co-infected plants. Therefore, the proteins allowing PAV transmission in *R. padi* differ from those in *S. avenae.*

The poleroviruses cereal yellow dwarf virus (CYDV; *Solemoviridae*) RPV and RMV also infect cereals causing barley yellow dwarf. Until recently, the genera *Polerovirus* and *Luteovirus* were assigned to the *Luteoviridae* family but have recently been reclassified as *Solemoviridae* and *Tombusviridae*, respectively. Despite having similar particle structures, sharing vectors and the transmission mechanism, no interactions between CYDV-RPV or RMV and BYDV-PAV were identified during their transmission, confirming that the transmission of these viruses relies on different proteins [62]. Indeed, the analysis of BYDV-PAV and CYDV-RPV transmission by a population of *Schizaphis graminum* Rondani (Hemiptera: Aphididae) determined that there was no genetic correlation between the ability to transmit BYDV-PAV and CYDV-RPV and that multiple loci are involved in the transmission of these viruses [65]. Even closely related poleroviruses, such as beet western yellows virus (BWYV) and cucurbit aphid-borne yellows virus (CABYV), do not share the same circulation route within the vector. These viruses have different gut tropism which is determined by their minor capsid protein [66].

Begomoviruses are also transmitted in a circulative manner but by whiteflies. Competition may occur depending on the similarity between the viruses. For example, pepper huasteco yellow vein virus (PHYVV) and pepper golden mosaic virus (PGMV) can be co-transmitted by *Bemisia tabaci* Gennadius (Hemiptera: Aleyrodidae) to pepper plants without competition [67]. Similarly, no competition was identified between two strains of tomato yellow leaf curl virus (TYLCV) in *B. tabaci* [68], but TYLCV and tomato mottle virus (ToMoV) competed when transmitted by *B. tabaci* [25]. Specific regions within the begomovirus coat protein are involved in the interaction with the gut and salivary gland receptors in the whitefly vectors [69,70,71].

The recent discovery of whitefly-transmitted poleroviruses [72,73] and of aphid-transmitted capulaviruses in the family *Geminiviridae* [74] has opened the door to analyze new interactions between more distantly related viruses with similar persistent circulative transmission mechanisms. The capulavirus alfalfa leaf curl virus (ALCV) and the nanovirus FBNSV infect broad bean plants and are transmitted by *Aphis craccivora* Koch (Hemiptera: Aphididae). Because capulaviruses and nanoviruses are structurally different, with no sequence homology between the proteins reported to bind the putative insect receptors, little interaction between these viruses is expected in plants and vectors. Indeed, they infect plants with little or no interference: their titers are not affected by co-infection, and they can co-infect the same cells. ALCV titer was higher than FBNSV in plants and in the insect gut [75]. However, the viruses accumulated at similar levels in the head of the insect, probably implying differences in their circulation and in the aphid proteins involved in their transmission. Immunolocalization of the viruses in the vector gut showed that ALCV and FBNSV could co-localize in the same midgut cells but in separate aggregates. Therefore, it is unlikely that they compete for aphid proteins in the gut. Indeed, the study of other geminiviruses and nanoviruses determined that while geminiviruses appear to use clathrin-mediated endocytosis to enter the vector gut [76], the nanoviruses might not [77]. It cannot be excluded that some common proteins involved in endocytosis, intracellular transport, or exocytosis are used by both viruses, in which case competition could occur even if the viruses bind to different receptors and accumulate in different aggregates within cells. Overall, that study illustrates that even in cases when viruses can co-infect plant cells and share similar circulation routes in their vector, they might not compete for vector proteins.

In conclusion, independently of their transmission mechanism, closely related viruses can compete during transmission, but that is not always the case. Virus transmission by insects is a complex phenomenon relying on several transient protein–protein interactions, and therefore the outcome of co-transmission appears to depend on the combination of viruses as well as the insect vector.

### 2.4. Co-Transmission Studies Involving Bacteria

Multiple bacterial pathogens or viruses and bacterial pathogens can co-infect plants [78]. For example, phytoplasmas can co-infect plants, and several surveys worldwide have identified phytoplasma and liberibacter bacteria co-infecting citrus trees [79,80]. Similarly, vectors such as the leafhoppers *Circulifer tenellus* Baker (Hemiptera: Cicadellidae) and *Dalbulus elimatus* Ball (Hemiptera: Cicadellidae) can carry and transmit phytoplasma and spiroplasma [81], or the psyllid *Bactericera cockerelli* (Šulc) (Hemiptera: Triozidae) can acquire and transmit different ‘*Candidatus* Liberibacter solanacearum’ haplotypes [82]. Several of these studies are reviewed in more detail [81,83,84]. Similarly, the establishment of ‘*Candidatus* Liberibacter asiaticus’ was reduced in orange trees previously infected with some CTV strains [85], and the bacterial pathogen had a more sporadic distribution within the co-infected plants. However, very few studies investigated in more detail the co-transmission of these pathogens and their potential interactions in the vector.

The ability of bacteria and persistent propagative viruses to replicate within the vector adds an additional factor that might affect transmission: these pathogens must not only interact with vector proteins and circulate through the vector body avoiding immune responses, but they must also replicate in the vector. Interactions affecting the ability of the pathogens to multiply in the vector because of competition or the induction of immune responses could affect the transmission. Indeed, for many of these pathogens, a minimum titer in the vector seems to correlate with transmission efficiency. For example, a correlation between the ability of psyllids to transmit ‘*Candidatus* Phytoplasma mali’ and its titer was established [86] and at least 10,000 ‘*Ca.* Liberibacter solanacearum’ in the salivary glands of the vector are needed for plant inoculation [87].

The study of the co-transmission of grapevine flavescence doree phytoplasma (FDP) and ‘*Ca.* Phytoplasma asteris’ chrysanthemum yellows strain (CYP) by nymphs of the leafhopper *Euscelidius variegatus* Kirschbaum (Hemiptera: Cicadellidae) determined that both bacteria could be sequentially acquired regardless of the feeding order, and the titer of FDP but not CYP in the vector were affected by the double infection [84]. While CYP was consistently and efficiently transmitted by co-infected leafhoppers, FDP was not. To evaluate if the competition occurred within the vector or following transmission to the host plant, the authors evaluated the bacterial titer in the salivary glands and in an artificial feeding medium. FDP was rarely detected in the salivary glands in the co-infected vectors and never in the artificial medium. Furthermore, a correlation between the CYP titer in the salivary glands and the pathogen transmission exists [88]. Therefore, CYP appears to outcompete FDP for the colonization of the vector salivary glands [83]. Interactions between vector-borne bacteria and several vector proteins potentially involved in the transmission process have been identified [89,90,91]; however, the competition among pathogens at the molecular level needs to be investigated.

## 3. Do Changes in the Pathogen Titer in the Host Affect the Co-Transmission?

Since a pathogen’s titer in a host plant can affect its transmission, if during co-infection the titer of a pathogen changes, its transmission could be affected. Indeed, correlations between changes in virus titers following co-infection and changes in transmission were identified in some pathosystems. For example, plant co-infection with cucurbit leaf crumple virus (CuLCrV, a begomovirus) and cucurbit yellow stunting disorder virus (CYSDV, a crinivirus) resulted in reduced CYSDV titer while CuLCrV titer was not affected [92]. Virus titers in the vector *B. tabaci* correlated with these changes: there were no differences in CuLCrV titer in the whiteflies, but the titer of CYSDV was reduced when the virus was acquired from co-infected plants. Because these viruses are not transmitted following the same mechanism and they share little structural similarity, it is expected that they interact with different vector components and therefore, competition between these pathogens during the transmission is unlikely. Whether the change in virus titer in the vector affected its transmission efficiency was not evaluated in the study.

It was also demonstrated that the changes in virus titer in the host and its effect on transmission could be host plant dependent. For example, tomato infectious chlorosis virus (TICV) titers increased during the co-infection of *Nicotiana benthamiana* plants with tomato chlorosis virus (ToCV) while ToCV titers decreased [93]. However, in *Physalis wrightii*, the titer of both criniviruses decreased during the co-infection, and ToCV accumulated to higher titers than TICV. These changes in virus titers were reflected in the transmission efficiency by *Trialeurodes vaporariorum* Westwood (Hemiptera: Aleyrodidae) when the viruses were acquired from co-infected plants. Further, in a different study, the co-infection of tomato plants did not affect the transmission of these two viruses by *T. vaporariorum* [94]. Based on these studies, the outcome of the competition between these viruses depends on the host plant. Further, it appears that the virus titer in the plant could correlate with the transmission efficiency. It is unclear if in these systems there was competition for *T. vaporariorum* proteins between these viruses. Indeed, competition for *T. vaporariorum* proteins cannot be excluded because these viruses are assigned to the same family [95]. However, these viruses might exploit different vector sites because some *B. tabaci* species can transmit ToCV but not TICV.

Several other studies demonstrated that a link between viral titer in the plant and the virus transmission efficiency is not always evident. For instance, the ability of aphids to transmit undetectable CTV strains is well established [96], and there is no correlation between the titer of PVY strains and their transmission efficiency [55].

Interesting results were obtained when studying the changes in viral titer of the closely related cucumber chlorotic yellows virus (CCYV) and cucurbit yellow stunting disorder virus (CYSDV) in co-infected plants. In this case, the changes in the viral titer did not correlate with changes in the transmission efficiency by *B. tabaci* MED [97]. Acquisition from co-infected plants resulted in a lower titer for each virus in the vector, but overall, the percentage of infectious whiteflies increased, and a higher percentage of infected plants were obtained following transmission by groups of insects. This increase in transmission efficiency could be linked to changes in the feeding behavior of the vector in co-infected plants. Indeed, plant infection can affect the behavior of the vector [97,98,99,100] leading to increased or reduced transmission independently of the effect in titer.

## 4. Co-Transmission: A Complex System without a Simple Solution

Vector acquisition of two pathogens does not ensure that both will be transmitted. The ability of each pathogen to be transmitted will depend on whether they are acquired from an infected host, can attach to their respective vector sites and persist long enough in the vector to be inoculated into a new host, are inoculated into a new host, and establish an infection. Another factor affecting the transmission of circulative pathogens is the efficiency with which they cross transmission barriers within the vector. Therefore, one single experiment evaluating disease development or pathogen titer in the recipient plant is not enough to understand the outcome of the co-transmission. Instead, a variety of approaches are needed as exemplified in some of the papers described above.

To evaluate and circumvent the potential competition in plants affecting pathogen acquisition several approaches can be used. One approach is to use co-infected plants as a pathogen source; however, if competition during transmission occurs, obtaining co-infected plants might be challenging. Depending on the pathosystem, this can be achieved by using mechanical inoculation [48], by using vectors exclusively carrying each pathogen [93], or when possible infecting the plant via other mechanisms such as agroinfiltration [32], biolistic delivery [67] or grafting [101]. These approaches do not necessarily preclude competition within the donor plant. Alternatively, when possible, acquisition can be achieved by insects feeding on artificial diets with purified virus particles [61] or cultured pathogens. A problem with the use of artificial diets is that the feeding behavior of the vector could be influenced, affecting in turn pathogen acquisition and thus transmission. Indeed, the host mounts defenses against the pathogens and also against the vector which can affect insect feeding [102]. Another issue that could arise is that the pathogen might depend on host components for acquisition and inoculation, in which case this approach is not well suited. Finally, sequential acquisition from single-infected plants can be performed. Several studies discussed here showed that the experimental setup used for the pathogen acquisition can influence the efficiency of its transmission and the occurrence of systemic infection in new hosts following inoculation: the transmission efficiency of a pathogen following sequential acquisition might be different than from co-infected plants [60]. Finally, the sequential acquisition of pathogens might affect the persistence of the first pathogen acquired [61,92]. Similar problems exist to evaluate if the pathogens were effectively transmitted: a reduction in the transmission efficiency of a pathogen could result from competition between pathogens in the vector or during the infection of the recipient plant. Artificial diets instead of recipient plants can be used to overcome some of these issues [25]; however, this approach has caveats as described above. Further, the use of artificial diets might affect the detachment of the pathogen from vector receptors. An alternative approach is the use of recipient plants that can only be infected by one of the pathogens [57], but this approach does not avoid the possibility of the plant mounting defenses against one pathogen, in turn affecting the other. Therefore, depending on the questions studied, several of these approaches might need to be used, and depending on the setup chosen, the obtained results might not be comparable between different studies. Therefore, as demonstrated by the study of a capulavirus and a nanovirus co-transmission [75], to evaluate competition within the vector, it might be necessary not only to measure pathogen accumulation in different organs of the vector, but also to visualize the pathogens within the vector to assess their distribution and colocalization. Finally, another issue that might arise when viruses co-infect plants is heterologous encapsidation, which occurs when the genomic material of one virus is totally or partially encapsidated by the coat proteins of another virus. In this case, the virus could be transmitted by a non-vector species [103]. Sequential acquisition experiments or the use of artificial diets for acquisition or inoculation could be used to avoid this problem.

## 5. Conclusions and Perspectives

Transmission is an essential step in a pathogen’s life cycle and is a key element of disease epidemiology. Co-infections can alter the disease phenotype and affect the vector fitness and behavior or the pathogen’s transmission efficiency by vectors. In nature, the co-infection of hosts does not rely exclusively on the co-transmission of pathogens as often several vectors feed on donor and recipient plants. The study of the co-transmission of pathogens can help elucidate the mechanisms involved in these processes. For instance, based on the studies discussed here it appears that virus titer in plants is not always correlated with transmission efficiency or that the interaction between viral proteins and nucleic acids might affect the conformity of the protein in turn affecting its ability to interact with vector proteins or the stability of protein–protein interactions. Pathogen transmission relies on a series of spatially and temporally controlled protein–protein interactions. Identifying key proteins involved in the transmission process and their interactions could lead to the development of tools to block transmission, screen vector populations, identify more transmissible pathogens, and overall improve our ability to manage diseases.

From an epidemiological perspective, the study of co-transmission only explains part of the results from the interaction of two pathogens. For example, it is possible that the ability of PVY^NTN^ to outcompete PVY^O^, and to be more likely transmitted and infect plants, might be in part associated with the increase in PVY^NTN^ and the decrease in PVY^O^ incidence in potatoes in the US. However, the net result of the competition between pathogens for transmission is only part of the picture that influences the disease epidemiology and spread of a specific pathogen [104]. Even if co-infection and/or co-transmission reduces a pathogen’s titer and its ability to infect a new host, the presence of another pathogen might induce changes in the vector behavior that can compensate for the costs associated with the co-infection and co-transmission [105]. The study of the co-transmission of CCYV and CYSDV by *B. tabaci* [97] discussed above is an example of how the changes in vector feeding behavior counter-balanced the reduced accumulation of the virus in the plant. A different study determined that while the co-infection of squash plants with ZYMV resulted in decreased accumulation of WMV in plants, the host changes associated with ZYMV infection, such as changes in leaf color and volatiles produced, increased the attraction of the vector *A. gossypii* [100]. Therefore, the effect of the host infection on the vector behavior needs to be included when evaluating co-transmission and disease epidemiology but very few studies do so.

Finally, another aspect that needs further study is the role of endosymbionts in the studied processes. The presence of endosymbionts can alter the vector competence: endosymbionts can produce proteins involved in the pathogen transmission, they can influence the tissue tropism of the pathogen in the vector, they can prime the vector immune response affecting the transmission of the pathogen, they can elicit plant responses affecting the vector behavior and consequently its ability to transmit pathogens, etc. [106,107,108,109]. Some vector-borne bacterial plant pathogens can also be considered insect endosymbionts, and as such, they can affect the transmission of other pathogens. Much less is known about molecular interactions occurring between vectors and bacterial pathogens than viral pathogens, as the former involves more proteins and often longer interactions. This lack of knowledge is evidenced by the paucity of systems in which the molecular interactions between bacterial pathogens and vectors were studied. Advancing the study of these interactions can help develop new approaches to manage the devastating diseases caused by these pathogens as well as to understand the role of bacteria in the biology of the vector and the transmission of other pathogens.

## Data Availability

Not applicable.
